# Mathematical and Machine Learning Approaches for Classification of Protein Secondary Structure Elements from *Cα* Coordinates

**DOI:** 10.3390/biom13060923

**Published:** 2023-05-31

**Authors:** Ali Sekmen, Kamal Al Nasr, Bahadir Bilgin, Ahmet Bugra Koku, Christopher Jones

**Affiliations:** 1 Department of Computer Science, Tennessee State University, Nashville, TN 37209, USA; asekmen@tnstate.edu (A.S.); bbilgin@tnstate.edu (B.B.); cjone141@tnstate.edu (C.J.); 2 Department of Mechanical Engineering, Middle East Technical University, Ankara 06800, Türkiye; kbugra@metu.edu.tr; 3 Center for Robotics and AI, Middle East Technical University, Ankara 06800, Türkiye

**Keywords:** protein structure modeling, protein secondary structure, secondary structure identification, machine learning, protein trace, mathematical modeling

## Abstract

Determining Secondary Structure Elements (SSEs) for any protein is crucial as an intermediate step for experimental tertiary structure determination. SSEs are identified using popular tools such as DSSP and STRIDE. These tools use atomic information to locate hydrogen bonds to identify SSEs. When some spatial atomic details are missing, locating SSEs becomes a hinder. To address the problem, when some atomic information is missing, three approaches for classifying SSE types using Cα atoms in protein chains were developed: (1) a mathematical approach, (2) a deep learning approach, and (3) an ensemble of five machine learning models. The proposed methods were compared against each other and with a state-of-the-art approach, PCASSO.

## 1. Introduction

Proteins form 3D structures, via atomic and molecular interactions, that determine their functions such as material or signal transporting, cell adhesion, and cell cycle [1,2]. Primary structures (sequences of amino acids in polypeptide chains) are known for a large set of proteins. However, only a small portion of them (<0.1%) have known tertiary structures (folding of a polypeptide chain into a 3D shape) and quaternary structures (special 3D arrangements of all polypeptide chains of a protein) via experimentation. Secondary structures (repeated patterns of folding of the protein backbone) are important to analyze relationship between primary and tertiary structures. Once the structure of a protein is determined, it is uploaded into a publicly available database such as Protein Data Bank (PDB) [3,4], which had 205 K proteins as of May 2023.

There are three experimental techniques used for determining 3D structures of proteins: X-ray crystallography [5,6,7], Nuclear Magnetic Resonance (NMR) spectroscopy [8,9], and Cryo-electron microscopy (Cryo-EM) [5,10,11].

- In a crystal, atoms and molecules arrange themselves in regular arrays and X-ray crystallography technology, which has been in use since the 1950s, utilizes this fact to generate atomic and molecular structure of the crystal. In order to determine the atomic structure of a protein, it first needs to be crystallized. However, protein crystallization is a difficult process and not possible for all proteins. For example, outer membrane proteins, mostly β-Barrel architectures, of Gram negative bacteria are mostly rigid and stable and therefore X-ray crystallography can be applied relatively easily to determine their molecular structures. However, high-resolution diffracting crystals of plasma membrane proteins and large molecules are not easy to crystallize, due to difficulty of obtaining homogeneous protein samples.
- NMR spectroscopy employs the properties of nuclear spin in the presence of an applied magnetic field to analyze the alignment of atoms’ nuclei and it also provides information about dynamic molecular interactions. NMR spectroscopy requires a large amount of pure samples and as with X-ray crystallography, it has difficulty analyzing molecules with large molecular weight.
- Cryo-EM provides a lower resolution view of a protein compared to X-ray crystallography. However, it does not require crystallization and therefore many proteins that are difficult to crystallize and large protein assemblies can be imaged using Cryo-EM. It creates a 3D image using thousands of 2D projections. Cryo-EM provides different level of views at near-atomic (<5 Å), subnanometer (5–10 Å), and nanometer (>10 Å), resolutions. Only near-atomic resolution can be used to identify locations of Cα and other atoms in the backbone of a protein chain.

It is known that the primary amino acid sequence for a protein chain includes all information to determine tertiary 3D structure of that chain. Computational modeling consists of several techniques to predict tertiary structure from primary structure [12,13,14,15,16]. Since it is computationally very heavy, it has mainly limited for smaller proteins (100–150 amino acids). Aplhabet/Google DeepMind recently developed the AlphaFold 2 AI system to predict tertiary structures with near experimental level accuracy [17]. There is another impactful machine learning approach for tertiary structure prediction called RosettaFold, as described in [18]. A review of several deep learning-based approaches can be found in [19]. In comparative or template-based modeling, the 3D structure of at least one protein is determined experimentally, this structure is then used to model other members of the same family of proteins based the alignment of the amino acid sequences [20,21].

Determining Secondary Structure Elements (SSEs) for any protein is crucial as an intermediate step for in vitro tertiary structure determination. SSEs are sub-conformational regions that form when a polypeptide chain folds because of some factors including hydrogen bonds between amino acid molecules. SSEs are commonly divided as helices (formed with hydrogen bonding of *N*-*H* and *C*=*O* groups four residues apart) and sheets (formed with hydrogen bonding of *N*-*H* group of one strand with *C*=*O* group of the adjacent strand). Any amino acid that is neither a helix nor sheet is categorized as a loop or coil. Experimentally, SSEs are located using optical measurements such as circular dichroism spectroscopy [22,23], infrared spectroscopy [24,25] and Raman spectroscopy or NMR chemical shifts [26,27].

A previous study [28] showed that approximately 40% of the protein structures deposited into database suffer from at least one or more missing backbone atoms, particularly, when higher resolution of the protein is not available. Further, the number of coarse grained proteins being constructed/simulated with Cα trace only is increasing. Therefore, assigning SSEs using Cα atoms only to tackle the problem of missing backbone atoms becomes a crucial step. Several approaches have been developed to determine the SSEs of protein using only the Cα atom locations. The first method used a sliding window covers four (4) consecutive residues to find the distances and dihedral angles of Cα atoms [29]. DEFINE relies on Cα coordinates only and compares Cα distances with distances in idealized secondary structure segments [30]. P-SEA assigns SSEs using a short Cα distance mask and two Cα dihedral angle criteria [31]. KAKSI uses Cα distance and backbone dihedral angles [32]. SACF identifies SSES based on the alignment of Cα backbone fragments with central poses derived by clustering known SSE fragment [33]. Other methods were developed to assign SSEs by approximating the backbone trace with a set of straight lines such as STICK [34] and PMML [35]. We have proposed a geometry-based approach using Cα trace that have reached 90% accuracy [36]. Recently, many machine learning approaches were developed. One example is the implementation of a neural network-based classifier called HECA [37]. HECA has two hidden layers, each with 128 neurons. It receives a set of rotational-invariant geometric features extracted from the raw coordinates of Cα atoms. In [33], an implementation of a classification algorithm called SACF (secondary structure assignment based on Cα fragments) is presented. In [38], a random forest classifier called RaFoSa is described for determining SSEs using a set of geometric features. We previously developed an ensembled machine learning approach using support vector machine (SVM), random forest (RF), Multilayer Perceptron (MLP), and XGBoost based on 20 geometric features [39]. In this paper, we use five different machine learning models with stacking and increase the number of geometric features. Subsequently, the accuracy is improved. In addition, a mathematical model and a deep learning model were developed based on 27 geometric features to tackle the problem [40,41]. In this paper, we use larger number of geometric features and extend/recast the methods to improve the accuracy. A comparison between the performance of the proposed mathematical models, deep learning model, the ensemble model, and a state of the art model is conducted with a large dataset in this paper.

This paper presents three approaches for classifying SSE types using Cα atoms only in protein chains. This is beneficial when atomic information is missing. A novel set of features are generated using locations and positioning of neighboring Cα atoms in a chain. The first approach is a mathematical approach that models each SSE as a subspace and the entire protein chain as a union of three subspaces. In this approach, a subspace is computed for each of the SSEs types α-helices, β-sheets, and loops. Unknown amino acids are classified based on two methods. In the first method, the distance from the amino acid’s feature vector to each subspace is computed. In the second method, a local subspace is matched for each amino acid and the subspace distances on the Grassmanian subspace manifold is computed. The second approach (Deep Learning) uses some categorical features in addition to the geometric features and employs two Network Architecture Search algorithms for selecting deep neural network architectures, layer connectives, and regularization parameters. The third approach (Ensemble of Machine Learning) stacks five models: Random Forest, Logistic Regression, k-Nearest Neighbor, Multilayer Perceptrons, and eXtreme Gradient Boosting.

## 2. Materials and Methods

### 2.1. Feature Generation

#### 2.1.1. Geometric Features

Our mathematical and machine learning models are based on geometrical features collected for the backbone of the protein structure, specifically, Cα trace (i.e., Cα coordinates). These geometrical features describe the geometry of each Cα atom and its surrounding neighborhood. For each Cα atom, we calculate a vector of geometric features, Fα, that consists of 39 features. Fα can be divided into seven categories of features. Each category is used to describe the geometry around Cα atom of interest in one aspect. Therefore, Fα = (Rα, Eα, Dα, Vα, Tα, Mα, Nα).

**Figure 1:**
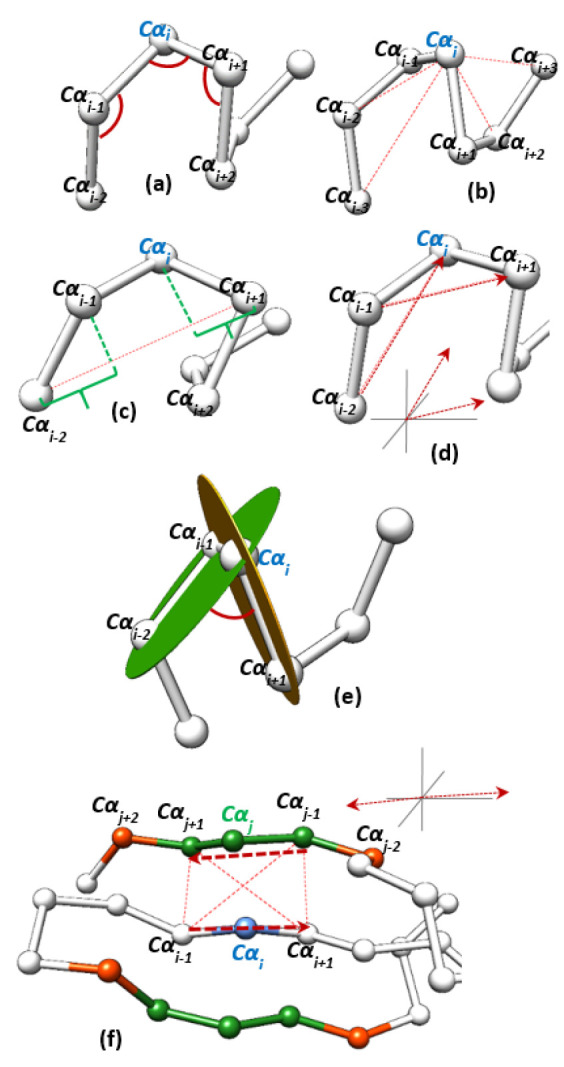
Geometric features calculated for a given $C\alpha_{i}$.

**Angle Features**, Rα. This category is used to calculate and to describe the geometric arrangements of Cα atoms around the Cα of interest, $C\alpha_{i}$. This category contains three different triangular angle values calculated around $C\alpha_{i}$. These angles are: angle(i−1,i,i+1), angle(i−2,i−1,i), and angle(i,i+1,i+2). Angle (i−1,i,i+1) is the interior angle centered at $C\alpha_{i}$ atom for the triangle formed between the three atoms ($C\alpha_{i-1}$, $C\alpha_{i}$, $C\alpha_{i+1}$). Similarly, angle(i−2,i−1,i) is the interior angle centered at $C\alpha_{i-1}$ atom for the triangle formed between the three atoms ($C\alpha_{i-2}$, $C\alpha_{i-1}$, $C\alpha_{i}$). The same idea is applied to calculate angle (i,i+1,i+2). Figure 1a shows an example of the three angles calculated around one $C\alpha_{i}$.

**Euclidean Distance Features**, Eα. This group of features is calculated by finding the Euclidean distance between the Cα atom of interest, $C\alpha_{i}$, and other Cαs in its region. It consists of four Euclidean distances: dist(i−3,i), dist(i−2,i), dist(i,i+2), and dist(i,i+3). Figure 1b shows an example of the four calculated distances around one $C\alpha_{i}$ in red dashed lines.

**Axis Distance Features**, Dα. This group of features is calculated by finding the distance between $C\alpha_{i}$ and other Cα atoms around it on virtual axes constructed in the surrounding region. It consists of eight values: axisDist(i−2,i−1), axisDist(i,i+1), axisDist2(i,i+1), axisDist(i−1,i), axisDist2(i−1,i), axisDist(i+1,i+2), axisDist(i−3,i−2), and axisDist(i+2,i+3). For instance, to calculate axisDist(i−2,i−1), we create a virtual axis connects $C\alpha_{i-2}$ and $C\alpha_{i+1}$ and the value of the distance is calculated between $C\alpha_{i-2}$ and the projection of $C\alpha_{i-1}$ on this virtual axis. Using the same virtual axis, we calculate axisDist(i,i+1) by finding the distance of the projection of $C\alpha_{i}$ and the coordinate of $C\alpha_{i+1}$. The idea is generalized to calculate all other axis distances. Each time a virtual axis is constructed and a distance is calculated by finding the distance between a Cα coordinate and a projection of another Cα coordinate or between the two projections of Cα coordinates such as in axisDist2(i,i+1). axisDist2(i,i+1) is calculated between the projections of $C\alpha_{i}$ and $C\alpha_{i+1}$ on the virtual axis constructed between $C\alpha_{i-2}$ and $C\alpha_{i+2}$. Figure 1c shows an example of axis distances axisDist(i−2,i−1) and axisDist(i,i+1) on the axis between $C\alpha_{i-2}$ and $C\alpha_{i+1}$.

**Vector Angle Features**, Vα. This group of features is calculated by finding the angles between some 3D vectors that are constructed around $C\alpha_{i}$. It contains four values: vAngle(i−2→i,i−1→i+1), vAngle(i−1→i+1,i→i+2), vAngle(i−3→i−1,i−2→i), vAngle(i→i+2,i+1→i+3). For instance, vAngle(i−2→i,i−1→i+1) is the angle between the vector that is constructed from the coordinates of $C\alpha_{i-2}$ and $C\alpha_{i}$ and the vector that is constructed between the coordinates of $C\alpha_{i-1}$ and $C\alpha_{i+1}$. The idea is the same for all other values in this category. Figure 1d shows the vector angle vAngle(i−2→i,i−1→i+1) and the angle between these two vectors is illustrated at the bottom.

**Torsion Angle Features**, Tα. The torsion angle is an example of a dihedral angle. It describes the geometric conformation and the relation of two parts of a molecule connected by a bond. It is the angle between two intersecting planes. Each plane is defined by three Cα coordinates. Therefore, the torsion angle can be calculated using four Cα coordinates. The first three coordinates define the first plane, and the last three coordinates define the second plane. This category consists of four torsion angle values: torsion(i−2,i−1,i,i+1), torsion(i−1,i,i+1,i+2), torsion(i−3,i−2,i−1,i), and torsion(i,i+1,i+2,i+3). As the definition suggests, each torsion angle is calculated by the coordinates of the four Cα atoms given. Figure 1e shows the torsion angle torsion(i−2,i−1,i,i+1).

**Miscellaneous Features**, Mα. This group of features contains some other features for each $C\alpha_{i}$. It contains five values: the amino acid type of the residue *i*, and summation of the four values of Vα and Tα. vAngle(i−2→i,i−1→i+1) is added to torsion(i−2,i−1,i,i+1), vAngle(i−1→i+1,i→i+2) is added to torsion(i−1,i,i+1,i+2), vAngle(i−3→i−1,i−2→i) is added to torsion(i−3,i−2,i−1,i), and vAngle(i→i+2,i+1→i+3) is added to torsion(i,i+1,i+2,i+3).

**Neighborhood Features**, Nα. This category of features is calculated to focus on the Cα coordinate of interest, $C\alpha_{i}$, and the shape and orientation of its surrounding. This category is the largest in terms of the number of values calculated. It consists of 11 values: four Euclidean distances, six scalar values, and one angular value for residue *i*. To calculate this group of features, we initially find a set of candidate neighbors of residue *i*. The surrounding is scanned and the atoms around $C\alpha_{i}$ are added to a candidates list. For residue *k*, it is added to the candidates list if it is at least three residues apart from residue *i* (i.e., i−2>k>i+2), the distance between *i* and *k* is less than 6.31Å, and there is another residue k’ in the candidates list that is adjacent to *k* such that $\left|{\mathrm{seqNum}}_{k}-{\mathrm{seqNum}}_{k}’\right|$ = 1. After the initial candidates list of neighbor residues is created, we keep only strong candidates in a final list. Residue *k* is added to the final list if the distance of $C\alpha_{k}$ and the line segment formed between residues i−1 and i+1 (i.e., $C\alpha_{i-1}$ and $C\alpha_{i+1}$) is less than 5.81Å and its projection is inside same line segment. These features are mainly used to describe the geometry surrounding of a residue on β-strands.

The features in $N\alpha_{i}$ are calculated using Cα atoms in the final list. After the final list of neighbors is created, six scalar values are calculated: the number of neighbors in the list, the length of the three eigenvectors of the point clouds formed by the Cα atoms in the list, the Euclidean distance of residue *i*, $C\alpha_{i}$, and residue *j*, $C\alpha_{j}$, where residue *j* is the closest residue to residue *i* from the list, and number of amino acids residue *i* and residue *j* are apart, $\mathrm{seqDiff}={\mathrm{seqNum}}_{i}-{\mathrm{seqNum}}_{j}$. Note that seqDiff could be a negative value if residue *j* comes after residue *i* in the sequence. Further, we calculate four Euclidean distances between *i*’s surrounding and *j*’s surrounding, where *j* is the closest residue in the neighbors list to residue *i*. These are the pairwise distances between $C\alpha_{i-1}-C\alpha_{j-1}$, $C\alpha_{i-1}-C\alpha_{j+1}$, $C\alpha_{i+1}-C\alpha_{j-1}$, and $C\alpha_{i+1}-C\alpha_{j+1}$. Finally, $N\alpha_{i}$ contains one angular value, which is the angle between the vector that is constructed from the coordinates $C\alpha_{i-1}$ and $C\alpha_{i+1}$ and the vector is constructed from the coordinates $C\alpha_{j-1}$ and $C\alpha_{j+1}$. Figure 1f shows an example of neighborhood features. In this example the first candidate list is found then the list is filtered to a final list. The Cα coordinates in red show some Cα atoms were in the initial candidate list and then removed from the final list. The Cα coordinates in green are examples of atoms make it to the final list. $C\alpha_{j}$ is the closest atom in the list to $C\alpha_{i}$ and the figure shows the four distances we calculate between atoms $C\alpha_{j-1}$, $C\alpha_{j+1}$, $C\alpha_{i-1}$, and $C\alpha_{i+1}$ in dashed lines. The two calculated vectors are shown and the angle between them is illustrated on the top right corner of Figure 1e.

#### 2.1.2. Determining Relevant Features

Given a feature matrix, reduction of features can be cast as in Problem 1, whose solution is provided in Algorithm 1. In this algorithm, a new rank estimation technique that was initially introduced in [40] is used. Some techniques such as in [42,43] were too sensitive and not very effective.

**Problem** **1.** *Let F be a d×N feature matrix whose columns represent Cα atoms where each atom has d feature points, i.e., N atoms in $R^{d}$.*

*1.*  *Determine k≤d, the number of most relevant features.*
*2.*  *Determine those k features.*

**  Algorithm 1: Reduction of features**.
**   Require: ***d* × *N* feature matrix **F**.
   1: Estimate effective-rank *k* of F (using rank estimation technique in [40]).
   2: Find a sub matrix with *k* rows and call it $F_{k}$.
   3: **while** effective-$\mathrm{rank}(F_{k})\neq k$ **do**
   4:     Find another sub matrix with *k* rows and call it $F_{k}$.
   5: 
**end while**
   6: *k* features corresponding to each row of $F_{k}$ are most relevant features.

### 2.2. Mathematical Approach

According to the Manifold assumption hypothesis, high-dimensional data in real world problems tend to lie in lower dimensional manifolds (or subspaces) [44]. For example, a set of face images (30 by 30 pixels) of a person with different facial expressions live in $R^{900}$ (ambient space) but they lie on a much lower dimensional manifold. It has been experimentally demonstrated that the face images of a person with the same facial expression under different illumination conditions lie on a 9 dimensional subspace of a very high dimensional ambient space [45]. It was also mathematically shown that trajectories of rigid body motions lie on 4 dimensional subspaces of a high dimensional ambient space [46,47,48]. In other words, many real world data may live in a high dimensional space $R^{n}$, but it typically comes from a union of *M* lower dimensional subspaces $S_{i}$ each coming from a subspace $R^{d_{i}}$, e.g., $U={⋃}_{i=1}^{M}S_{i}$ where $d_{i}<n$ is the dimension of subspace $S_{i}$. In this research, Cα traces are grouped in a sliding-window so that each group represents a data point in a high-dimensional ambient space. Then, a lower dimensional subspace is matched to data points of each SSE type. When a group of unknown Cα traces is presented, a neighborhood of each Cα is determined and a local subspace is matched to Cα traces inside each neighborhood. Then, the separation of this local subspace from each SSE subspace is computed using geodesic distance on the Grassmannian manifold of the subspaces [49]. A simpler subspace projection approach is also developed for computing distance between a data point in ambient space and each SSE subspace.

#### 2.2.1. SSE Subspace Modeling

**Figure 2:**
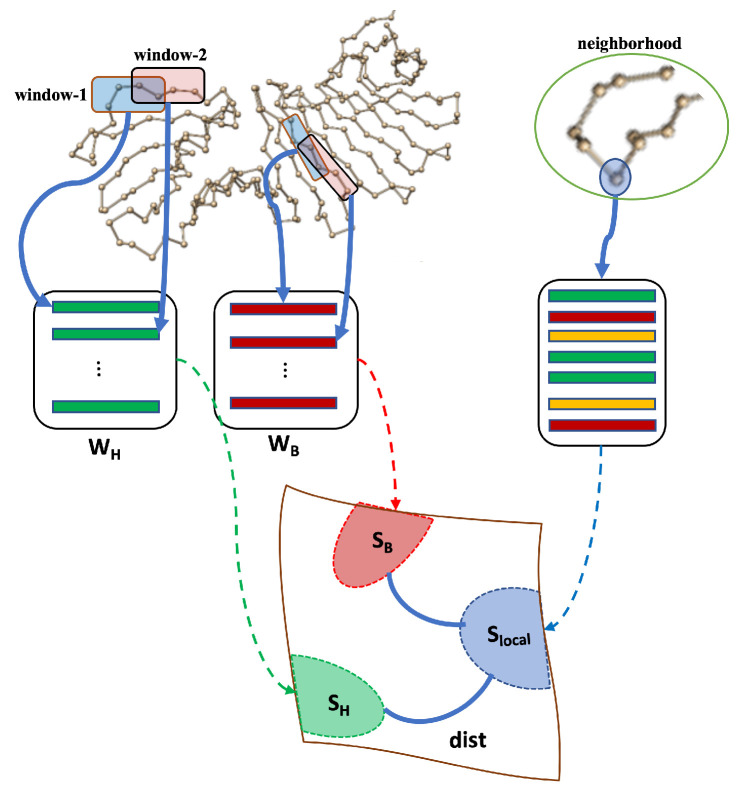
Subspace modeling with local subspace matching and separation.

Each SSE and intersecting region is represented with a subspace. A sliding-window approach is developed for representing each Cα in the training set as a high-dimensional data point (Figure 2). The problem can be cast as in Problem 2 and a solution is provided in Algorithm 2.

**Problem** **2.** *Let each Cα atom have d geometric features, i.e., $C\alpha \left(i\right)\in R^{d}$ for all $i\leq N_{a}$, where $N_{a}$ is the number of amino acids in the training protein. Assume that the window size is q. In that case, each window includes q atoms with Cα(i) in the center. Determine a subspace for each SSE type. $S_{H}$, $S_{B}$, and $S_{L}$, representing the subspaces for helices, sheets, and loops, respectively.*

First, a data matrix for each SSE and intersecting region is constructed by concatenating *d* features for each of *q* atoms as a single column vector for a single window. Then, the next window is formed by sliding it by a certain number of atoms and the second column is constructed. If the new window is in another SSE, then the column is moved into the corresponding data matrix. Finally, Singular Value Decomposition (SVD) is used to match a suitable subspace for each data matrix.
**  Algorithm 2: SSE subspace matching**.
**   Require: ***q*: window size, *z*: window-sliding size, $N_{a}$: the number of amino acids.   1: Create empty data matrices $W_{H}$, $W_{B}$, and $W_{L}$.   2: Form first possible window.   3: **for all **
$i\leq N_{a}$
** do**   4:      Form (qd)×1 column vector $w_{i}={\left[C\alpha (i-q/2+1)\cdots C\alpha (i)\cdots C\alpha (i+q/2)\right]}^{T}$.   5:      Expand the corresponding data matrix by adding $w_{i}$ as a new column vector.   6:      Slide the window by *z*.   7: **end for**   8: **for all** data matrices **do**   9:      Compute SVD. For example, $W_{H}=U_{H}\Sigma_{H}V_{H}^{T}$.       10:      Estimate the rank (using rank estimation technique in [40]). For example, $\;\;\;\;\;\;\;\;\;\;\;\;\;\;\;\;\mathrm{rank}\left(W_{H}\right)=r_{H}$.       11:      Compute a subspace. For example, $S_{H}=\mathrm{Span}\left(u_{H_{1}},\cdots ,u_{H_{r_{H}}}\right)$, i.e., the span                  of the first $r_{H}$ columns of $U_{H}$.       12: **end for**


#### 2.2.2. Projections on SSE Subspaces and Classification

In order to classify a Cα atom in a test protein, a group of other Cα atoms in its neighborhood is identified. Then, two approaches are adopted. A subspace for each SSE has already been determined in the previous section. For example, $W_{H}=U_{H}\Sigma_{H}V_{H}^{T}$ and $S_{H}$ is the subspace spanned by the first $r_{H}$ columns of $U_{H}$, where $r_{H}$ is the effective rank of $W_{H}$. In this case,

- Let ${\tilde{U}}_{H}=U_{H}\left(1:r_{H}\right)$ be $U_{H}$ with truncation after the first $r_{H}$ columns.
- Let $w_{i}\in R^{qd}$ be the data vector for the Cα(i) that is being classified. Note that, the same window size as in the training is used.

The distance between $w_{i}$ and $S_{H}$ is computed simply by projecting $w_{i}$ onto $S_{H}$:

(1) $$d_{i}={∥\left(I-{\tilde{U}}_{H}{\tilde{U}}_{H}^{T}\right)w_{i}∥}_{2}$$

Then, Cα(i) is classified based to the shortest distance to all SSE subspaces.

#### 2.2.3. Local Subspaces and Classification

In this approach, a local subspace is generated to represent the Cα(i) atom that is being classified. In order to do this,

- A group of neighbors of Cα(i) is identified as illustrated in Figure 2. Each Cα atom in the neighborhood is in $R^{qd}$ using the same window size as before.
- Let $N_{l}$ be the number of atoms in each neighborhood.
- Construct $\left(qd\right)\times N_{l}$ matrix whose columns are representation of each Cα in the neighborhood. Call this matrix L.
- Compute SVD of $L=U_{l}\Sigma_{l}V_{l}^{T}$.
- Let $S_{local}$ be the local subspace spanned by the columns of $U_{l}$.

Cα(i) is classified based on the separation of $S_{local}$ from SSE subspaces. There are different measures for separation of subspaces. Each subspace can be represented as a point in a Grassmannian manifold [49] and various distances such as geodesic arc length, chordal distance, or projection distance can be considered. In this work, the chordal distance is used as follows:

(2) $$d=\sum_{j=1}^{p}sin^{2}\theta_{j}$$

where $\theta_{1},\theta_{2},\cdots ,\theta_{p}$ are the principal angles between two subspaces. In order to find the principal angles between $S_{local}$ and $S_{H}$, orthonormal bases $Q_{l}$ and $Q_{H}$ are first computed using SVD. Then, a new matrix $Q=Q_{l}^{T}Q_{H}$. Let $1\geq \sigma_{1}\geq \sigma_{2}\geq \cdots \geq \sigma_{p}\geq 0$ be the singular values of *Q*, then the principal angles are given by

(3) $$\theta_{k}=\arccos\left(\sigma_{k}\right)\;\;\;\;k=1,\cdots ,p.$$

All distances between the local subspace and each SSE subspace are calculated and then, Cα(i) is classified based to the shortest distance.

#### 2.2.4. Post Processing

To improve the accuracy of the classifier for some residues, we apply a post processing step. This post processing step is important to classify residues with missing Fα vector or portion of it due to a missing Cα coordinate. Further, this step is used for residues that are classified as one of the three SSEs and it is isolated where the entire surrounding is of a different type. For example, one residue is classified as helix while the rest of residues before and after are classified as loop. This could occur if the distance of residue subspace with helix subspace and loop subspace is too close to each other.

We start to correct the classification of incorrect helix classification and change it to either sheet or loop based on Nα features. If the number of neighbors for the residue is more than two and it is classified as helix, it is more likely that this is a wrong classification. Therefore, we check the number of distance values in Nα. If the number of distance values in Nα that are less than 6 is less than three, it is changed to loop; otherwise, it is changed to sheet because it shows a high compactness in the region which denote a strand region.

Similarly, we change the classification of some residues from loop to sheet if the number of neighbors in Nα is greater than four and the number of distance values in Nα that are less than 6 is greater than two. On the other hand, we change it to helix if the number of neighbors in Nα is less than three and the number of distance values that are less than 6 is five. Finally, if a residue is isolated inside a group of residues from another type, we change its classification to match the type of the group.

### 2.3. Deep Learning Approach

#### 2.3.1. Dataset

In this study, a dataset of 3946 proteins which consist of 904,081 amino acids and their 39 features is used. Out of the 39 features utilized in this study for SSE classification, one feature corresponds to the name of the amino acid. In order to convert a qualitative feature element, such as the name of an amino acid, into a quantitative one so that they can be used in training, one-hot-encoded representation is used. Given that there are 20 amino acids, the name element in the feature vector is replaced with a one-hot-encoded vector of length 20 resulting in a feature vector of size 58. This dataset contains the ground truth values as SSE types which are also labels, i.e., α-helices, β-sheets and loops, as explained in Section I. The labels of the SSE types are similarly one-hot-encoded into a label vector of size 3. The dataset is divided into training-validation-test sets of 70%−20%−10% of protein chains, respectively. 37 angle and distance based features inside sets are standardized (μ=0, σ=1) using the standard deviation and mean obtained from the training set.

Protein chains consist of n×57 feature vectors representing *n* amino acids. Each protein chain is padded with 3×57 empty matrices on both ends. A rolling window with size 7 is shifted on each protein chain resulting in *n* input feature matrices with size 7×57. Each 7×57 input matrix is checked for empty valued features (non existing features). One 7×57 matrix with ones for non-empty features and zeros for empty features is created. Similarly, another 7×57 matrix with zeros for non-empty features and ones for empty features is created. Then, empty features inside the input matrix are replaced with zeros. Finally, all three matrices are stacked and a tensor with size 7×57×3 is obtained. Each input tensor is used to predict a single SSE element. The amino acid of interest sits at fourth row of this tensor. To summarize, when generating the input vector, not only the amino acid of interest, but also the neighboring amino acids are considered. Furthermore, empty features in the input vector are taken into account. As a result, accuracy is improved with respect to the previous work [41].

#### 2.3.2. Network Architecture and Training Parameters

A deep neural network architecture is used in this work. This deep neural network architecture takes the flattened 7×57×3 tensor in the shape of 855 input neurons and runs it through a series of fully connected layers. In our previous work [41], a neural architecture search (NAS) algorithm is utilized to select hyper-parameters such as batch size and number of hidden layers. The NAS algorithm creates and trains neural networks by selecting hyper-parameters from a search space. Then, the hyper-parameters with the best neural network performance are selected. In this work, the hyper-parameters selected by the neural architecture search algorithm from previous work are used. The network uses categorical cross-entropy as a loss function and ADAM as optimization method for training. The learning rate is reduced automatically while training as evaluation measures stop improving for multiple epochs until a certain threshold is reached.

#### 2.3.3. Evaluation Measures

After SSE output vectors are obtained for amino acids, the highest probability SSE type is used to label a particular amino acid. As a result, evaluation measure is selected as categorical accuracy.

#### 2.3.4. Joint Prediction of Multiple Architectures

Deep neural networks are typically applied for non-convex problems such as SSE classification. Because of random initialization of the network weights, each training results in different network performance with possible convergence to a different local minima. If network hyper-parameters are properly tuned for a sufficiently complex network architecture, a smoother loss surface is expected for optimization. In other words, each training of neural network is expected to have similar performance. However, this does not necessarily mean each network correctly labels the same set of samples. For this reason, multiple neural network architecture predictions can be “joint” (ensembled) to give their predictions together given an input vector. These prediction probabilities can be added together and their maximum can be selected as the correct label. In most cases this results in higher accuracy and robustness in comparison to using a single trained network as shown in Table 1. Multiple neural networks trained for this method are same in all aspects except the number of neurons. The number of neurons are selected from a small range around the values obtained by NAS algorithm in previous work [41]. The accuracy of the multiple neural networks trained using deep learning approach and the accuracy obtained by joint prediction of neural network approaches are explained in the Section 3.

### 2.4. Ensemble of Machine Learning Models Approach (EML)

In this approach, five machine learning (ML) models with stacking to assign SSEs using the geometry of Cα trace are used. The method assigns one of the standard SSE types (Helix, Sheet, Loop) for each of the residues in a given protein. The ML models used initially are, Random Forest (RF), Logistic Regression (LR), K-Nearest Neighbor (KNN), Multilayer Perceptron (MLP), and eXtreme Gradient Boosting (XGBoost). The approach uses the set of geometric features collected for each Cα atom, Fα. The ensemble model goes through the following steps: data set determination, data preparation and cleaning, training, fine tuning, and stacking. The result is a 3-state classifier.

**Data set and cleaning:** We used the list of protein chains in cullpdb_pc20_res1.8_R0.25_d200528_chains5510 from PISCES server [50] to build our data set. The list contains 5510 PDB chains in total with the maximum R-factor of 0.25, resolution of 1.8 Å or better and sequence identity of 20% or less. We excluded any protein with the following: PDB chains missing information for SSEs, PDB chains includes a code of insertion, PDB chains include unknown amino acids, and PDB chains missing any Cα coordinates. After cleaning, a total number of 3946 PDB files/chains remained in the candidate list and it is called set I. The total number of residues in set I is approximately 868 K. Note that the total number of cleaned residues in the dataset is different than the total number of residues in Deep Learning approach since the two approaches use different methods to clean data. Set I was divided into two sets, T and S. Set S consists of 300 proteins (i.e., 69,491 residues) for testing and the rest of proteins were maintained in set T. Set T contains approximately 799 K. A set of 600 K residues (i.e., 200 K from each SSE type) is chosen randomly from set T to train our ML ensemble model. T is divided into two sets, set Tr for training consists of 480 K (i.e., 80% of T) and set Ts consists of 120 K (i.e., 20% of T) for individual model evaluation and testing.

**Preparation:** When the distribution of the geometric features for Tr, Fα, are plotted, nine features out of the 39 had skewed distribution (data not shown). Therefore, the set of geometric features were preprocessed by standardization with quantile transformation [51] and selected with the best *k* method. The features were standardized with a quantile transformation with 200 bins, which makes each transformed feature to have a Gaussian distribution. After standardization, we applied a best *k* features selection method by ANOVA F-value between 1 and 39. Selecting the best *k* features was conducted by two stages. In stage one, the best combinations of *k* features are selected. 39 feature combinations were generated, one for each *k* value from 1 to 39. In stage two, each of the 39 combinations was evaluated by 10-fold cross-validation. Accuracy was used as the metric for the evaluation. The feature combination with the best performance was used in training a model. For our SSE identifier model, we choose the entire 39 features, and therefore, k=39.

**Training:** Five classification models were selected with default parameters: Random Forest (RF), Logistic Regression (LR), K-Nearest Neighbor (KNN), Multilayer Perceptron (MLP) 0.23 machine learning package and XGBoost model was implemented with XGBoost Python module. Both accuracy and f1 scores were used as the evaluation metrics. Each ML model was evaluated with 10-fold cross-validation. The RF and XGBoost models have better accuracy and f1 scores than the other two models MLP. The accuracies were: RF (95.6%), LR (86.4%), KNN (90.2%), MLP (91.5%), and XGBoost (93.2%). F1 scores were: RF (95.6%), LR (86.4%), KNN (90.1%), MLP (91.4%), and XGBoost (93.2%). We chose to proceed with the highest four models for the following steps, fine tuning and stacking.

**Fine Tuning and Stacking:** The four ML models were further fine-tuned to search the best parameter combinations using the grid search with 10-fold cross-validation. The best parameters for each model were selected. For example, an RF model with 1500 trees and five maximum features, MLP model with one 50-neural hidden layer, XGBoost model with learning rate eta = 0.2, max depth = 6, and 300 trees were generated as the fine-tuned models. Other parameters uses the default values. At the end, the four fine-tuned models were ensembled with a stacking approach in which the outputs of the four models were used as the inputs to a logistic regression model with default parameters [52]. The models trained from the stacking were delivered as the final models.

## 3. Results

To evaluate the performance of our models, we validate each one individually. The three models (i.e., Mathematical Subspace, Deep Learning, and EML), which all use the same dataset (i.e., PISCES dataset), are trained and validated individually. In this section, we will report the evaluation of the models separately and at the end of the section, we will test all models against one of the existing ML approaches in the literature (i.e., PCASSO) using the same benchmark.

### 3.1. Results: Subspace Segmentation Approach

The performance of these two models was evaluated using set S (i.e., 300 proteins randomly chosen). This test is used to compare the performance of these models individually and with other models.

For a detailed analysis for the performance of these classifiers on S dataset, we include the performance table and confusion matrix in Table 2, Table 3, Table 4 and Table 5. The total number of residues tested is 68,572. For Model-1 (i.e., distance-based subspace model), Table 3 shows that the true positive are 26,681, 12,022, and 18,809 for helices, sheets, and loops, respectively. Our classifier was able to assign 26,681 out of 31,521 residues to helix class correctly. Relative to the total residue in each class, we see that the helix class was the class with the highest true positive (i.e., 84.6%) and loop class was the lowest with true positive (i.e., 82.9%). This is expected since the geometric shape of the loop is flexible and irregular. Further, the helix class is the class with the fewest false positive cases (i.e., 1009 cases). On the other hand, sheet is the class with the fewest false negative cases (i.e., 2351). For Model-2 (i.e., local-subspace model), Table 5 shows that the true positive for helix class was 26,082 out of 31,521, was 11,043 out of 14,373 for sheet class, and was 16,803 out of 22,678 for loop class. Again, the helix class is the best performing class and the worst performed class is the loop class. For false positive cases, the helix class is again the best performing with only 1236 cases. Compared to each other, we find that Model-1 performs better than Model-2. The accuracy of Model-1 is 83.9% and Model-2 is 78.6% as shown in Table 2 and Table 4.

### 3.2. Results: Deep Learning Approach

The performance of this model is evaluated using two sets of test data, The first set is the 10% of training data which consists of 410 protein chains. For this set, joint prediction accuracy is 95.08%. The other set (set S) is 300 proteins selected from these 410 protein chains, which is used as a common test set by all approaches in this paper. Firstly; the accuracy for joint prediction for S data is given in Table 6. Secondly; Precision score, recall, F1 score, and accuracy are calculated for the joint prediction model on S data which can be seen in Table 7.

### 3.3. Results: Ensemble of Machine Learning Models

The performance of this model was evaluated using two sets of test data. The first set is Ts (i.e., 20% of training data) as explained above. Ts consists of 120 K (i.e., 40 K from each type) randomly selected Cα atoms (i.e., residue level). The other set is S is our common test set which is used to compare the performance of this model with other models. Ts is used to evaluate the performance of this 3-state classifier individually.

We used four metrics to report the performance of this model for Ts dataset in Table 8. The metrics are precision score, recall, F1 score, and accuracy. The model is a 3-state classification model; therefore, its precision, recall, and F1 contains three numbers representing the scores for helix, sheet, and loop. The F1 scores are 97%, 97%, and 95% for helix, sheet, and loop, respectively. It shows that our classifier has a similar performance on classifying helix and sheet SSEs and a slightly worse ability on assigning loop residues. The total accuracy of the system (i.e., 96.3%) shows that this classifier is able to correctly classify the three secondary structure elements well.

For a detailed analysis for the performance of our classifier on Ts dataset, we include the confusion matrix in Table 9. The total number of residues in the test is 120 K (i.e., 40 K from each class). The table shows that the highest true positive class was sheet class. Our classifier was able to assign 38,895 out of 40,000 residues to sheet class correctly. The helix class show a similar level of true positive and finally is loop class is classified with less accuracy. This is expected based on the flexible shape of loops. The helix class is the class with the fewest false positive cases (i.e., 1087 cases). On the other hand, the sheet class presents the fewest false negative cases (i.e., 1105). Again, helix class performance is very similar to the sheet class with 1111 false negative cases. From Table 9, we can conclude that the performance of helix class and sheet class is comparable and loop class comes at the end. In addition, we conclude that the classifier confuses the most between loop residues and sheet residues. for instance, there are 1088 sheet residues were predicted as loops and 1129 loops residues predicted as sheets. The classifier is much more successful at differentiating between helix and sheet classes.

We used the same four metrics to report the performance of this model for S dataset (i.e., our 300 protein common test set) in Table 10. The F1 scores are 96%, 93%, and 90% for helix, sheet, and loop, respectively. It shows that our classifier performed better on helix class than the other two classes and demonstrates a slightly worse ability on assigning sheet and loop residues. The total accuracy of the system (i.e., 93.51%) shows that this classifier performed slightly worse on S dataset than on Ts dataset.

For a detailed analysis for the performance of our classifier on S dataset, we include the confusion matrix in Table 11. The total number of residues in the test is 65,996. The table supports the results of the classifier with Ts data. The performance of the system on helix and sheet data is better than on loop data. This is expected since the geometry of helix is easier to detect. Further, the neighborhood features helped to distinguish many sheet residues. Note: that the number of residues processed in each of our developed model in this paper is slightly different because the way each model processes and cleans data is different.

### 3.4. Results: Existing Approach

As a comparison for accuracy we include the results in Table 12 and Table 13, on the same set S of 300 proteins, generated by PCASSO [28]. To generate our comparison PCASSO was used to generate SSE predictions for each of the proteins in dataset S. Those predictions were then compared to the SSEs as documented in the PDB of each protein.

PCASSO is a well established, widely used tool for predicting SSEs based on Cα traces. PCASSO uses a set of geometric features derived from Cα locations and from calculated pseudocenter location based on the geometric center of C$\alpha_{i}$ and C$\alpha_{i+1}$. PCASSO develops 43 feature per Cα and by combining these features has 258 features available per location. PCASSO uses 16 of these available feature and a RF with 50 trees to make its predictions. A confusion matrix for PCASSO is presented in Table 13.

### 3.5. Results: Summary

To compare the performance of developed classifiers in this paper to each other and with one of the exiting methods in the literature (i.e., PCASSO), we evaluated the performance using a unified dataset, S. S consists of 300 randomly chosen proteins. Each protein has a sequence identity of 20% or less with any protein in the set used to train our classifier (i.e., set T). The data set is comprised of high-resolution protein molecules (i.e., resolution range: 0.92 Å to 1.75 Å). Further, the data set contains a variety of protein sizes. The smallest protein is made up of 12 residues (1T7M chain B) and the largest protein is made up of 1053 (6DT6 chain A) residues and the average size of the proteins in this set is 228.57 residues. The output of each classifier is compared with the SSEs assignment from PDB file for each protein. If a residue assignment matches the residue assignment in PDB file, it is a hit; otherwise, it is a miss. The accuracy is the total number of hits over the total number of residues in the protein. Table 14 reports the accuracy of all classifiers on these proteins (i.e., S dataset). From the table, we can conclude that DL approach is the best performer approach on this dataset followed by the EML. The worst performer is Subspace model II. PCASSO was ranked the third approach in the list.

To show the performance of the models on some of the proteins in dataset S, we chose 30 random proteins from S. The performance of the models on these proteins is shown in Table 15. We chose 10 proteins where the models do not perform well, 10 other proteins where the models perform well, and 10 other proteins where the models perform on average. The average size of selected proteins is 131.33 residues. The largest protein is 5DWD (PDB ID) chain D with 481 residues and the smallest protein is 3SSB (PDB ID) chain I with 30 residues.

## 4. Discussion

Three-dimensional structure is a key for understanding the biological function of a protein. Therefore, several experimental (i.e., X-ray crystallography and Cryo-EM) and computational techniques (i.e., ab initio and comparative) are used to determine the tertiary structure of a protein. One crucial step in determining the structure of a protein is determining the secondary structure elements (SSEs). SSEs are sub-conformational regions that form when a polypeptide chain folds because of some factors including hydrogen bonds between amino acids. SSEs can be categorized into three types: helices, β-sheets, and loops/coils. Computationally, SSEs are determined using pattern recognition process of hydrogen-bonds and geometrical features extracted from full-atom protein coordinates. The most popular methods are DSSP and STRIDE. When a group of atoms is missing structural data (i.e., coordinate), conventional methods such as DSSP will not perform as intended.

In this research, we present a multi-model approach for identifying SSEs using Cα trace only. This mimics a scenario of proteins when atomic information is missing. Our approach consists of two mathematical models (Model-I and Model-II), one deep learning model (DL), and one ensemble of machine learning model (EML). All models use a set of 39 geometric features collected for each amino acid describing its neighborhood geometrically using Cα coordinates only. A set of 5510 predetermined proteins (i.e., 868K amino acids) was used to extract these features and train our models. A large set, set S, that consists of 300 proteins and 69K amino acids was used to validate our models and compare it with a state-of-the-art approach, PCASSO. The experimental comparison has shown that DL model has the best performance on set S with accuracy reached 95.12%. EML model was ranked second with accuracy reaching 93.51%. On the other hand, PCASSO and Model-I were ranked at the bottom of the list.

## Figures and Tables

**Table 1:** Neural Network accuracy on validation set.

Network ID	Validation Accuracy
#1	94.12%
#2	94.14%
#3	94.17%
#4	94.13%
#5	94.24%
#6	94.24%
#7	94.11%
#8	94.15%
#9	94.27%
#10	93.88%
Joint Prediction	95.06%

**Table 2:** The performance of Model-1 approach on S data.

	Precision	Recall	F1-Score	Accuracy
Helix	96.4%	84.6%	90.1%	83.9%
Sheet	79.2%	83.6%	81.3%	
Loop	73.2%	82.9%	77.7%	

**Table 3:** The confusion matrix for Subspace Model-1 for S data.

Observed/Predicted	Helix	Sheet	Loop	Total
Helix	26,681 (84.65%)	286 (0.90%)	4554 (14.45%)	31,521
Sheet	8 (0.06%)	12,022 (83.64%)	2343 (16.30%)	14,373
Loop	1001 (4.41%)	2868 (12.65%)	18,809 (82.94%)	22,678
Total	27,690	15,176	25,706	68,572

**Table 4:** The performance of Model-2 approach on S data.

	Precision	Recall	F1-Score	Accuracy
Helix	95.4%	82.7%	88.6%	78.6%
Sheet	69.2%	76.8%	72.8%	
Loop	66.4%	74.1%	70.0%	

**Table 5:** The confusion matrix for Subspace Model-2 for S data.

Observed/Predicted	Helix	Sheet	Loop	Total
Helix	26,082 (82.74%)	248 (0.79%)	5191 (16.47%)	31,521
Sheet	26 (0.18%)	11,043 (76.83%)	3304 (22.99%)	14,373
Loop	1210 (5.34%)	4665 (20.57%)	16,803 (74.09%)	22,678
Total	27,318	15,956	25,298	68,572

**Table 6:** The performance of deep learning approach on S data.

	Precision	Recall	F1-Score	Accuracy
Helix	98.09%	97.33%	97.70%	95.12%
Sheet	92.35%	93.88%	93.11%	
Loop	92.74%	92.79%	92.77%	

**Table 7:** The confusion matrix for deep learning approach for S data.

Observed/Predicted	Helix	Sheet	Loop	Total
Helix	30,918 (98.09%)	24 (0.07%)	579 (1.84%)	31,521
Sheet	44 (0.31%)	13,274 (92.35%)	1055 (7.34%)	14,373
Loop	804 (3.55%)	842 (3.71%)	21,032 (92.74%)	22,678
Total	31,766	14,140	22,666	68,572

**Table 8:** The performance of EML classifier on Ts data.

	Precision	Recall	F1-Score	Accuracy
Helix	97%	97%	97%	96.3%
Sheet	97%	97%	97%	
Loop	95%	95%	95%	

**Table 9:** The confusion matrix for EML classifier for Ts data.

Observed/Predicted	Helix	Sheet	Loop	Total
Helix	38,889 (97.22%)	24 (0.06%)	1087 (2.72%)	40,000
Sheet	17 (0.04%)	38,895 (97.24%)	1088 (2.72%)	40,000
Loop	1070 (2.67%)	1129 (2.82%)	37,801 (94.50%)	40,000
Total	39,976	40,048	39,976	120,000

**Table 10:** The performance of EML classifier on S data.

	Precision	Recall	F1-Score	Accuracy
Helix	97%	96%	96%	93.51%
Sheet	92%	93%	93%	
Loop	89%	90%	90%	

**Table 11:** The confusion matrix for EML classifier for S data.

Observed/Predicted	Helix	Sheet	Loop	Total
Helix	29,654 (95.85%)	26 (0.08%)	1259 (4.07%)	30,939
Sheet	15 (0.11%)	13,064 (92.93%)	979 (6.96%)	14,058
Loop	935 (4.45%)	1066 (5.08%)	18,998 (90.47%)	20,999
Total	30,604	14,156	21,236	65,996

**Table 12:** The performance of PCASSO approach on S data.

	Precision	Recall	F1-Score	Accuracy
Helix	98.3%	77.7%	86.8%	84.3%
Sheet	87.1%	86.4%	86.7%	
Loop	69.8%	92.8%	79.7%	

**Table 13:** The confusion matrix for PCASSO.

Observed/Predicted	Helix	Sheet	Loop	Total
Helix	24,702 (77.67%)	349 (1.10%)	6751 (21.23%)	31,802
Sheet	12 (0.09%)	12,233 (86.35%)	1921 (13.56%)	14,166
Loop	413 (1.92%)	1145 (5.31%)	20,001 (92.77%)	21,559
Total	25,127	14,036	28,673	67,526

**Table 14:** The accuracy of all models on S dataset.

Dataset	EML	Model-1	Model-2	DL	PCASSO
S	93.51	83.9	78.6	95.12	84.3

**Table 15:** The performance of all models on test data.

Num	Protein ID ^a^	Chain ID ^b^	#AA ^c^	EML% ^d^	Subspace I% ^e^	Subspace II% ^f^	DL% ^g^	PCASSO% ^h^
1	3SSB	I	30	75.0	83.9	78.6	80.0	26.7
2	2END	A	137	75.6	83.8	78.7	76.6	81.8
3	1ZUU	A	56	80.0	84.0	78.8	78.6	80.4
4	4UE8	B	37	63.3	83.7	78.5	67.6	48.6
5	5W82	A	100	77.4	84.0	78.8	77.0	94.0
6	3QR7	A	115	80.7	84.1	78.8	73.9	95.2
7	3NGG	A	46	82.5	83.9	78.8	87.0	84.4
8	3X34	A	87	81.3	84.1	78.8	83.9	63.3
9	1KVE	A	63	75.5	83.9	78.7	73.0	90.5
10	5DBL	A	130	86.3	84.0	78.7	94.6	66.7
11	4KK7	A	385	93.7	83.7	78.5	94.0	88.8
12	3MAO	A	105	91.9	84.0	78.8	95.2	87.6
13	5QS9	A	171	95.2	83.8	78.7	96.0	90.6
14	6DWD	D	481	94.2	83.5	78.4	96.7	85.2
15	4G9S	B	111	98.1	83.6	78.7	98.2	30.1
16	3ZVS	A	158	94.7	83.9	78.7	96.2	96.6
17	3QL9	A	125	93.3	84.0	78.7	93.6	87.9
18	4ZFL	A	229	94.1	83.8	78.7	93.4	86.7
19	5OBY	A	365	94.1	83.5	78.5	96.7	86.1
20	6B1K	A	114	93.3	84.2	78.8	90.4	85.1
21	4ONR	A	147	100	84.0	78.8	99.3	93.8
22	2IC6	A	71	100	84.1	78.8	100	94.4
23	3HE5	B	48	100	84.2	78.9	97.9	95.7
24	3LDC	A	82	100	84.0	78.8	100	91.5
25	4ABM	A	79	100	83.8	78.8	100	94.9
26	4I6R	A	77	100	84.0	78.7	100	62.0
27	4WZX	A	87	100	84.0	78.8	98.9	87.2
28	5OI7	A	88	100	83.9	78.5	98.9	95.4
29	3D3B	A	139	100	84.2	78.8	100	84.2
30	5XAU	B	71	100	84.1	78.8	98.6	97.2
Average			131.13	90.7	83.9	78.7	91.2	81.8

^a^: Protein ID (4 letter/digit) as in PDB; ^b^: The ID of the chain used in the experiment; ^c^: The total number of Amino acids in the chain; ^d^: The percentage accuracy of EML approach; ^e^: The percentage accuracy of Subspace Model I; ^f^: The percentage accuracy of Subspace Model II; ^g^: The percentage accuracy of ML approach; ^h^: The percentage accuracy of PCASSO tool.

## Data Availability

Protein chains in cullpdb_pc20_res1.8_R0.25_d200528_chains5510 from PISCES server [50] are used to build our data set. Further details are provided in Section 2.3.1 and Section 2.4.

## References

[1] Ridley M. (2000). Genome.

[2] Murray R.K., Granner D.K., Mayes P.A., Rodwell V.W. (2006). Harper’s Illustrated Biochemistry.

[3] Burley S.K., Berman H.M., Bhikadiya C., Bi C., Chen L., Di Costanzo L., Christie C., Dalenberg K., Duarte J.M., Dutta S. (2018). RCSB Protein Data Bank: Biological macromolecular structures enabling research and education in fundamental biology, biomedicine, biotechnology and energy. Nucleic Acids Res..

[4] Sussman J.L., Lin D., Jiang J., Manning N.O., Prilusky J., Ritter O., Abola E.E. (1998). Protein Data Bank (PDB): Database of three-dimensional structural information of biological macromolecules. Acta Crystallogr. Sect. D Biol. Crystallogr..

[5] Tarry M.J., Haque A.S., Bui K.H., Schmeing T.M. (2017). X-Ray Crystallography and Electron Microscopy of Cross- and Multi-Module Nonribosomal Peptide Synthetase Proteins Reveal a Flexible Architecture. Structure.

[6] Tsai C., Schertler G.F.X. (2020). Membrane Protein Crystallization.

[7] Maveyraud L., Mourey L. (2020). Protein X-ray Crystallography and Drug Discovery. Molecules.

[8] Hatzakis E. (2019). Nuclear Magnetic Resonance (NMR) Spectroscopy in Food Science: A Comprehensive Review. Compr. Rev. Food Sci. Food Saf..

[9] Li W., Zhang Y., Skolnick J. (2004). Application of sparse NMR restraints to large-scale protein structure prediction. Biophys J..

[10] Danev R., Yanagisawa H., Kikkawa M. (2019). Cryo-Electron Microscopy Methodology: Current Aspects and Future Directions. Trends Biochem. Sci..

[11] Wrapp D., Wang N., Corbett K.S., Goldsmith J.A., Hsieh C.L., Abiona O., Graham B.S., McLellan J.S. (2020). Cryo-EM structure of the 2019-nCoV spike in the prefusion conformation. Science.

[12] Terashi G., Kihara D. (2018). De novo main-chain modeling for EM maps using MAINMAST. Nat. Commun..

[13] Chen M., Baldwin P.R., Ludtke S.J., Baker M.L. (2016). De Novo modeling in cryo-EM density maps with Pathwalking. J. Struct. Biol..

[14] Al Nasr K., Chen L., Si D., Ranjan D., Zubair M., He J. Building the Initial Chain of the Proteins through de Novo Modeling of the Cryo-Electron Microscopy Volume Data at the Medium Resolutions. Proceedings of the BCB ’12 ACM Conference on Bioinformatics, Computational Biology and Biomedicine.

[15] Al Nasr K. (2012). De Novo Protein Structure Modeling from Cryoem Data through a Dynamic Programming Algorithm in the Secondary Structure Topology Graph. Ph.D. Dissertation.

[16] Al Nasr K., He J. (2016). Constrained cyclic coordinate descent for cryo-EM images at medium resolutions: Beyond the protein loop closure problem. Robotica.

[17] Senior A.W., Evans R., Jumper J., Kirkpatrick J., Sifre L., Green T., Qin C., Zidek A., Nelson A.W.R., Hassabis D. (2020). Improved protein structure prediction using potentials from deep learning. Nature.

[18] Baek M., DiMaio F., Anishchenko I., Dauparas J., Ovchinnikov S., Lee G.R., Wang J., Cong Q., Kinch L.N., Schaeffer R.D. (2021). Accurate prediction of protein structures and interactions using a three-track neural network. Science.

[19] Pakhrin S.C., Shrestha B., Adhikari B., Kc D.B. (2021). Deep Learning-Based Advances in Protein Structure Prediction. Int. J. Mol. Sci..

[20] Lam S.D., Das S., Sillitoe I., Orengo C. (2017). An overview of comparative modelling and resources dedicated to large-scale modelling of genome sequences. Acta Crystallogr. Sect. D.

[21] Pandit S.B., Zhang Y., Skolnick J. (2006). TASSER-Lite: An automated tool for protein comparative modeling. Biophys J..

[22] Greenfield N.J. (1996). Methods to Estimate the Conformation of Proteins and Polypeptides from Circular Dichroism Data. Anal. Biochem..

[23] Provencher S.W., Gloeckner J. (1981). Estimation of globular protein secondary structure from circular dichroism. Biochemistry.

[24] Dousseau F., Pezolet M. (1990). Determination of the secondary structure content of proteins in aqueous solutions from their amide I and amide II infrared bands. Comparison between classical and partial least-squares methods. Biochemistry.

[25] Byler D.M., Susi H. (1986). Examination of the secondary structure of proteins by deconvolved FTIR spectra. Biopolymers.

[26] Wishart D.S., Sykes B.D., Richards F.M. (1992). The chemical shift index: A fast and simple method for the assignment of protein secondary structure through NMR spectroscopy. Biochemistry.

[27] Pastore A., Saudek V. (1990). The relationship between chemical shift and secondary structure in proteins. J. Magn. Reson..

[28] Law S.M., Frank A.T., Brooks C.L. (2014). PCASSO: A fast and efficient *Cα*-based method for accurately assigning protein secondary structure elements. J. Comput. Chem..

[29] Levitt M., Greer J. (1977). Automatic identification of secondary structure in globular proteins. J. Mol. Biol..

[30] Richards F.M., Kundrot C.E. (1988). Identification of structural motifs from protein coordinate data: Secondary structure and first-level supersecondary structure. Proteins Struct. Funct. Bioinform..

[31] Labesse G., Colloc’h N., Pothier J., Mornon J.P. (1997). P-SEA: A new efficient assignment of secondary structure from *Cα* trace of proteins. Bioinformatics.

[32] Martin J., Letellier G., Marin A., Taly J.F., de Brevern A.G., Gibrat J.F. (2005). Protein secondary structure assignment revisited: A detailed analysis of different assignment methods. BMC Struct. Biol..

[33] Cao C., Wang G., Liu A., Xu S., Wang L., Zou S. (2016). A New Secondary Structure Assignment Algorithm Using *Cα* Backbone Fragments. Int. J. Mol. Sci..

[34] Taylor W.R. (2001). Defining linear segments in protein structure. J. Mol. Biol..

[35] Konagurthu A.S., Allison L., Stuckey P.J., Lesk A.M. (2011). Piecewise linear approximation of protein structures using the principle of minimum message length. Bioinformatics.

[36] Si D., Ji S., Al Nasr K., He J. (2012). A machine learning approach for the identification of protein secondary structure elements from cryoEM density maps. Biopolymers.

[37] Saqib M.N., Kryś J.D., Gront D. (2022). Automated Protein Secondary Structure Assignment from *Cα* Positions Using Neural Networks. Biomolecules.

[38] Salawu E.O. (2016). RaFoSA: Random forests secondary structure assignment for coarse-grained and all-atom protein systems. Cogent Biol..

[39] Sallal M.A., Chen W., Al Nasr K. Machine Learning Approach to Assign Protein Secondary Structure Elements from *Cα* Trace. Proceedings of the 2020 IEEE International Conference on Bioinformatics and Biomedicine (BIBM).

[40] Sekmen A., Al Nasr K., Jones C. Subspace Modeling for Classification of Protein Secondary Structure Elements from *Cα* Trace. Proceedings of the IEEE International Conference on Bioinformatics and Biomedicine (BIBM).

[41] Al Nasr K., Sekmen A., Bilgin B., Jones C., Koku A.B. Deep Learning for Assignment of Protein Secondary Structure Elements from *Cα* Coordinates. Proceedings of the IEEE International Conference on Bioinformatics and Biomedicine (BIBM).

[42] Vidal R., Ma Y., Sastry S. (2005). Generalized Principal Component Analysis (GPCA). IEEE Trans. Pattern Anal. Mach. Intell..

[43] Roy O., Vetterli M. The effective rank: A measure of effective dimensionality. Proceedings of the 2007 15th European Signal Processing Conference.

[44] Berner J., Grohs P., Kutyniok G., Petersen P. (2021). The modern mathematics of deep learning. arXiv.

[45] Ho J., Yang M., Lim J., Kriegman D. Clustering appearances of objects under varying illumination conditions. Proceedings of the IEEE Conference on Computer Vision and Pattern Recognition.

[46] Aldroubi A., Sekmen A. (2012). Nearness to local subspace algorithm for subspace and motion segmentation. IEEE Signal Process. Lett..

[47] Vidal R. (2010). A tutorial on subspace clustering. IEEE Signal Process. Mag..

[48] Georghiades A.S., Belhumeur P.N., Kriegman D.J. (2001). From Few to Many: Illumination Cone Models for Face Recognition under Variable Lighting and Pose. IEEE Trans. Pattern Anal. Mach. Intell..

[49] Zhang J., Zhu G., Heath R.W., Huang K. (2018). Grassmannian Learning: Embedding Geometry Awareness in Shallow and Deep Learning. arXiv.

[50] Wang G., Dunbrack R.L. (2003). PISCES: A protein sequence culling server. Bioinformatics.

[51] Bolstad B., Irizarry R., Åstrand M., Speed T. (2003). A comparison of normalization methods for high density oligonucleotide array data based on variance and bias. Bioinformatics.

[52] Wolpert D.H. (1992). Stacked generalization. Neural Netw..

